# Factors Associated with Difficult-to-Treat Rheumatoid Arthritis (D2T-RA): Real-World Evidence from a Single-Center Cross-Sectional Study

**DOI:** 10.3390/jpm16020065

**Published:** 2026-01-29

**Authors:** Maurizio Benucci, Francesca Li Gobbi, Emanuele Antonio Maria Cassarà, Riccardo Terenzi, Elisa Cioffi, Christian D’Elia, Sabrina Aliberti, Serena Guiducci, Edda Russo, Barbara Lari, Valentina Grossi, Maria Infantino, Mariangela Manfredi

**Affiliations:** 1 Rheumatology Unit, S. Giovanni di Dio Hospital, Azienda USL Toscana Centro, 50143 Florence, Italy; maurizio.benucci@uslcentro.toscana.it (M.B.); emanueleantonio.cassara@uslcentro.toscana.it (E.A.M.C.); riccardo.terenzi@uslcentro.toscana.it (R.T.); elisa.cioffi@uslcentro.toscana.it (E.C.); 2 Rheumatology Division, Department of Experimental and Clinical Medicine, University of Florence, 50121 Florence, Italy; christian.delia@unifi.it (C.D.); sabrina.aliberti@unifi.it (S.A.); serena.guiducci@unifi.it (S.G.); 3 Clinical Pathology S. Giuseppe Hospital, Azienda USL Toscana Centro, Empoli, 50143 Florence, Italy; edda.russo@uslcentro.toscana.it; 4 Immunology and Allergology Laboratory Unit, S. Giovanni di Dio Hospital, Azienda USL Toscana Centro, 50143 Florence, Italy; barbara.lari@uslcentro.toscana.it (B.L.); valentina2.grossi@uslcentro.toscana.it (V.G.); maria2.infantino@uslcentro.toscana.it (M.I.); mariangela.manfredi@uslcentro.toscana.it (M.M.)

**Keywords:** difficult to treat, rheumatoid arthritis, b-DMARDs, ts-DMARDs

## Abstract

**Background**: Rheumatoid arthritis (RA) is a chronic, systemic autoimmune disease characterized by persistent synovial inflammation and progressive joint destruction. Despite the implementation of the treat-to-target (T2T) strategy and the introduction of several classes of biologic and targeted synthetic disease-modifying antirheumatic drugs (b/tsDMARDs), a considerable proportion of patients continues to exhibit active, refractory disease. In 2021, the European Alliance of Associations for Rheumatology (EULAR) defined this condition as Difficult-to-Treat Rheumatoid Arthritis (D2T-RA). This study aimed to identify clinical, laboratory, and therapeutic factors associated with D2T-RA. **Methods**: A total of 344 patients with established RA were retrospectively evaluated. Among them, 164 fulfilled the 2021 EULAR criteria for D2T-RA (D2T group), while 180 did not (NO-D2T group). Clinical (age, sex, disease duration, BMI, smoking, comorbidities), laboratory (RF, ACPA, ESR, CRP), clinimetric (DAS28, CDAI, PhGA, PGA, HAQ), and therapeutic data (glucocorticoid use, methotrexate treatment and dose, monotherapy, advanced therapy exposure, number of failed advanced therapies, current DMARD regimen) were analyzed. **Results**: Factors significantly associated with D2T-RA included female sex, longer disease duration, higher RF and ACPA titers, elevated ESR levels, glucocorticoid therapy, and a greater number of failed advanced therapies. Although both groups achieved low disease activity or remission by DAS28 and CDAI, JAK inhibitors—particularly Filgotinib and Upadacitinib—were significantly more common in the D2T cohort and appeared associated with clinical stabilization. **Conclusions**: This study strengthens the understanding of the predictive profile of D2T-RA, confirming the role of disease chronicity and persistent inflammation in the development of treatment resistance. Importantly, the observed trend toward clinical stabilization achieved under JAK inhibitor therapy reinforces their potential to address unmet therapeutic needs in D2T-RA, providing a mechanistically grounded strategy for patients refractory to conventional and biologic DMARDs.

## 1. Introduction

Difficult-to-treat Rheumatoid Arthritis (D2T-RA) has been defined by a task force of the European Alliance of Associations for Rheumatology (EULAR) [1] based on the fulfillment of three main criteria: (1) a history of treatment failure with at least two biological or targeted synthetic disease-modifying antirheumatic drugs (b/tsDMARDs) with distinct mechanisms of action—such as tumor necrosis factor inhibitors (anti-TNF), interleukin-6 inhibitors (anti-IL-6), co-stimulation modulators (e.g., abatacept), B-cell depleting agents (rituximab), or Janus kinase inhibitors (JAK inhibitors); (2) evidence of persistent active or symptomatic disease, as indicated by a Disease Activity Score in 28 joints (DAS28) > 3.2 or a Clinical Disease Activity Index (CDAI) > 10, inability to taper glucocorticoids below 7.5 mg/day of prednisone, rapid radiographic progression; and/or (3) the perception—by either the patient or the physician—of difficulty in managing the disease.

The estimated prevalence of D2T-RA varies considerably across different cohorts, ranging from approximately 5% to 20% [2,3,4,5]. This variability may reflect multiple contributing factors [6], including differences in the timing and intensity of pharmacologic intervention [7]. However, it remains unclear whether D2T-RA represents a distinct clinical phenotype with an as-yet unidentified pathogenic substrate present from the early stages of disease, or whether it emerges as a consequence of disease progression and therapeutic history. The risk factors predisposing individuals to develop D2T-RA are not yet fully elucidated. However, several features have been proposed, including high disease activity at onset and the presence of high-titer rheumatoid factors (RFs) or anti-citrullinated protein antibodies (ACPAs)—a triad often associated with poor prognosis [8].

Moreover, comorbidities are present in up to 70% of D2T-RA cases and may significantly affect both disease assessment and therapeutic decision-making [9]. These comorbid conditions may be pre-existing, arise as complications of long-standing disease and chronic inflammation, or result from prolonged glucocorticoid exposure—all of which can contribute to worse outcomes and more complex management [10]. Therefore, a comprehensive understanding of the impact of comorbidities on the clinical profile of RA patients, particularly in those who develop D2T-RA, is essential [11,12,13]. In our single-center, cross-sectional analysis of RA patients using real-world data from our prescription database, we identified a D2T-RA prevalence of 34.3% [14]. Therefore, the primary objective of this investigation was to assess clinical, laboratory, and therapeutic factors associated with progression to D2T-RA, with the aim of identifying the most relevant factors contributing to its development within our patient cohort.

## 2. Methods

### 2.1. Study Population

A total of 344 patients diagnosed with RA, comprising 271 females and 73 males, were enrolled in this study. All patients fulfilled the 2010 American College of Rheumatology/European Alliance of Associations for Rheumatology (ACR/EULAR) classification criteria for RA [15] and were under active follow-up during the year 2025 at the Rheumatology Unit of San Giovanni di Dio Hospital-USL Toscana Centro. Patients were stratified into two subgroups based on whether they met the 2021 EULAR criteria for D2T-RA [1]. According to this classification, out of the 344 patients (100%), 180 (52%) were classified as non-D2T (NO-D2T), while 164 patients (48%) met the criteria for D2T-RA. The determination of PGA and PhGA completed the criteria for the patient’s and physician’s assessment of the state of dissatisfaction with the health status

### 2.2. Inclusion Criteria

Patients were included if they met the following criteria at the time of data collection in June 2025:

Confirmed diagnosis of RA;

Presence of at least one comorbidity, including arterial hypertension, cardiovascular disease, diabetes mellitus, fibromyalgia, depression, interstitial lung disease, chronic obstructive pulmonary disease (COPD), osteoporosis, or chronic kidney disease (CKD);

Current or past treatment with conventional synthetic DMARDs (csDMARDs), biological DMARDs (bDMARDs), or targeted synthetic DMARDs (tsDMARDs).

### 2.3. Data Collection and Study Design

This was a retrospective, observational study. Data were obtained through

Review of patients’ electronic medical records;

Extraction of clinical data from an existing hospital database;

Collection of clinical and laboratory parameters from the most recent outpatient follow-up visit.

### 2.4. Collected Variables

The following data were recorded:

Demographic variables: name and surname, age, sex;

Clinical and anamnestic data: Disease duration (expressed in months from time of diagnosis), smoking status (current smoker, former smoker, never smoked), body mass index (BMI, kg/m^2^), presence of comorbidities (arterial hypertension, cardiovascular disease, fibromyalgia, depression, interstitial lung disease, COPD, osteoporosis, CKD, diabetes mellitus).

Therapeutic data: Current DMARD therapy (csDMARDs, bDMARDs, tsDMARDs), use of monotherapy or combination therapy (specifically methotrexate co-medication and weekly dosage), daily dose of prednisone, and number of prior failures with bDMARDs or tsDMARDs. Furthermore, the percentage of patients treated with prednisone and methotrexate was evaluated.

Clinimetric assessments at the last visit: Disease Activity Score in 28 joints (DAS28-ESR), Clinical Disease Activity Index (CDAI), number of tender and swollen joints (28-joint count), Patient Global Assessment (PGA), Physician Global Assessment (PhGA), Health Assessment Questionnaire (HAQ), and Visual Analogue Scale (VAS).

Laboratory parameters at the last visit: Erythrocyte sedimentation rate (ESR), C-reactive protein (CRP), rheumatoid factor (RF) titer (N Latex RF; Siemens AG, Munich, Germany), and anti-cyclic citrullinated peptide antibody (ACPA) levels (EliA CCP; Phadia AB, Uppsala, Sweden).

### 2.5. Statistical Analysis

Descriptive statistics were reported as percentages (%) to illustrate the incidence of categorical variables. Given the non-normal distribution of the data, continuous variables were summarized using the median and IQR. For comparisons of categorical variables between groups, Fisher’s exact test was employed. For comparisons of medians between independent groups, the Mann–Whitney U test was used. A two-tailed *p*-value of less than 0.05 was considered statistically significant. All statistical analyses were conducted using MedCalc Statistical Software version 23.0 (© 2023 MedCalc Software Ltd., Acacialaan 22, 8400 Ostend, Belgium).

## 3. Results

Table 1 presents the baseline demographic, clinical, and therapeutic characteristics of the study population, along with statistically significant differences identified between patients classified as no-D2T and those meeting the criteria for D2T.

Statistical analysis demonstrated a significant difference in sex distribution between the two study cohorts. Male sex was significantly less prevalent in the D2T group (13%; 21/164) compared to the NO-D2T cohort (29%; 52/180) (*p* = 0.004). Conversely, no statistically significant difference was found for female sex distribution between groups (71%; 128/180 in NO-D2T vs. 87%; 143/164 in D2T; *p* = NS).

Disease duration, expressed as months since the diagnosis of RA, was markedly longer in D2T patients, with a median [IQR] of 108 [84–120] months, compared to 60 [20–114] months in the NO-D2T cohort (*p* < 0.0001). Moreover, data dispersion was narrower in D2T patients, indicating a more homogeneous disease duration distribution.

Among serological variables, RF levels were significantly higher in the D2T group (median [IQR]: 64 [20–156] IU/mL) than in the NO-D2T group (46 [20–98] IU/mL; *p* = 0.031). Similarly, anti-citrullinated protein antibody (ACPA/anti-CCP) levels were significantly elevated in D2T patients (median [IQR]: 178 [20–1600] IU/mL) compared to NO-D2T patients (108 [20–360] IU/mL; *p* = 0.0053).

Inflammatory activity, as reflected by the ESR, also differed significantly between groups (*p* = 0.018). Indeed, the D2T population showed a higher median [IQR] ESR value of 18 [13–20] mm/h, which was approximately double that of the NO-D2T group (9 [6–19] mm/h).

Additionally, corticosteroid use was significantly more frequent in D2T patients (17%; 28/164) than in NO-D2T patients (8.8%; 16/180; *p* = 0.028).

Likewise, advanced therapy use (including biologic DMARDs [bDMARDs] and targeted synthetic DMARDs [tsDMARDs]) was substantially higher among D2T patients, involving nearly all subjects (99.3%; 163/164) versus 75% (135/180) in the NO-D2T group (*p* < 0.0001).

**Figure 1:**
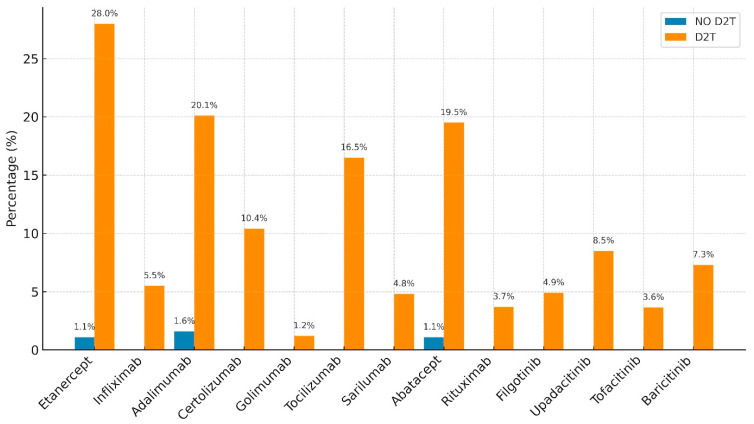
Treatment differences with b-DMARDs and ts-DMARDs.

Table 2 and Figure 1 summarize the distribution of individual advanced therapies across the two groups, showing a clear predominance of advanced agents in the D2T cohort.

**Figure 2:**
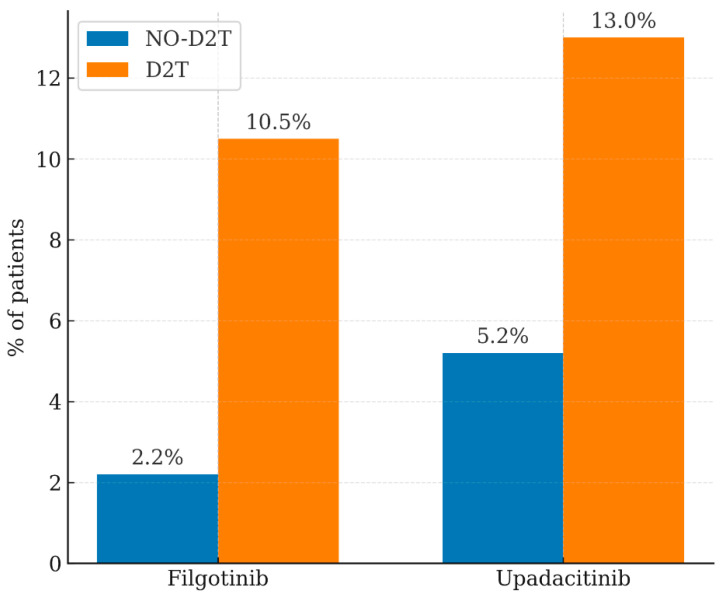
Percentage of JAK inhibitor use in the D2T and NO-D2T populations: Filgotinib (*p* = 0.015) and Upadacitinib (*p* = 0.0053) (see Table 3).

Notably, two JAK inhibitors—Filgotinib and Upadacitinib—showed statistically significant between-group differences, being more frequently used in D2T patients (10.5% vs. 2.2%, *p* = 0.015, and 13.0% vs. 5.2%, *p* = 0.0053, respectively; Figure 2). For all other biologic or targeted synthetic DMARDs, including Tocilizumab, Etanercept, and Abatacept, no statistically significant difference was detected between groups (Table 3). Regarding treatment persistence, the study retrospectively evaluated the last 18 months (January 2024–June 2025).

No statistically significant differences were detected between the two patient cohorts with regard to other comorbidities or clinimetric measures.

Given the inter-relationship between clinical, serological, and therapeutic variables, we performed two multivariable analyses: logistic regression and multiple regression, to reinforce the robustness of the findings. For both tests, we utilized the “classification as D2T” as the dependent variable, and the covariates are all the clinical and therapeutic characteristics recorded in Table 4 and Table 5. We reported only the data resulted predictor. About the missing data, we decided to apply the pairwise deletion. In both evaluations, four parameters represent the predictors of disease progression to D2T-RA, and the value of R^2^ confirms the reliability of the model. In the following table, we have only reported the significant values. Female gender, a longer disease duration, the highest ACPA titer, and the highest ESR value lead to the development of a difficult-to-treat RA.

## 4. Discussion

In our study, cross-sectional analysis of the D2T-RA population highlighted several distinctive characteristics. Male sex was found to be less prevalent in the D2T group (13% of the total, 21/164 patients) compared to the NO-D2T group (29%, 52/180 patients). These findings suggest that male sex may be associated with a lower likelihood of progression to D2T-RA, potentially conferring a protective effect in the clinical course of the disease. However, female sex remained predominant in both study populations (D2T and NO-D2T). Our results are consistent with previous studies reporting female sex as the most prevalent among patients with D2T-RA and associating it with multiple bDMARD resistance and poorer remission outcomes [16,17,18]. Further supporting evidence indicates that women experience worse disease progression—despite comparable treatments—during follow-up, as reflected by higher DAS28-ESR and HAQ scores, whereas men more frequently achieve clinical remission [19]. In agreement, another study identified male sex as an independent predictor of disease remission, while female sex was associated with radiographic progression [20].

Moreover, disease duration was significantly longer in the D2T group (median 108 months, IQR = 84–120) compared to the NO-D2T group (median 60 months, IQR = 20–114). This finding aligns with previous studies on large RA cohorts, including one with 1709 patients (173 D2T-RA, 10.1%), where female sex, low body weight, advanced age, and prolonged disease duration were all associated with D2T-RA [21]. Similar associations were confirmed by the KOBYO registry, which identified D2T-RA in 11.7% of 2321 patients treated with b/tsDMARDs [22], and by an Italian cohort of 458 patients in which disease duration was significantly longer in the D2T-RA subgroup (median 15 years *vs*. 10 years) [23]. In addition, elevated RF titers were observed in the D2T group. This finding is consistent with the KURAMA cohort study, which identified high RF levels as an unfavorable prognostic factor associated with increased disease activity, greater joint damage, and progression toward D2T-RA. Therefore, baseline RF levels should be carefully considered in RA patients to identify individuals at risk of developing D2T-RA and to guide early, personalized therapeutic strategies [4]. Similar evidence supports the role of RF in the pathogenetic mechanisms of D2T-RA and links high RF titers to disease refractoriness [24]. Moreover, the association between high RF levels and type I interferon suggests a possible contribution to treatment resistance in early RA [25,26]. Moreover, ACPA titers were also more prevalent in the D2T group. Our data indicate that elevated ACPA concentrations correlate with a greater likelihood of progression to D2T-RA, supporting their role as predictive biomarkers of difficult-to-treat disease, consistent with previous studies [27,28]. Elevated ESR values were also more frequent in the D2T population. These results suggest that higher ESR values may serve as risk indicators for progression to D2T-RA. Supporting this interpretation, Khader et al. (2022) demonstrated that an ESR ≥ 20 mm/h is a significant negative predictor of remission following biologic therapy [18]. The association between corticosteroid therapy and D2T-RA identified in our analysis is consistent with previous studies comparing D2T-RA with easy-to-treat (E2T-RA) populations. Interestingly, factors associated with D2T-RA included high disease activity (DAS28-ESR), altered gut microbiota composition (reduced *Firmicutes*/*Bacteroidetes* ratio), and corticosteroid use [29]. Another study reported that, in addition to high disease activity, comorbidities, pain, disability, and greater DMARD use, higher glucocorticoid doses represented negative predictive factors for disease evolution [30]. In our study, nearly all patients in the D2T group were treated with advanced therapies (99.3%, 163/164) compared to 75% (135/180) in the NO-D2T group. This finding aligns with the 2021 EULAR criterion 1 for D2T-RA diagnosis [1] and corroborates previous literature indicating a greater prevalence of b/tsDMARD use among D2T-RA patients [30]. A statistically significant difference emerged for the use of JAK inhibitors, specifically Filgotinib (10.5% of D2T vs. 2.2% of NO-D2T patients; *p* = 0.015) and Upadacitinib (13% vs. 5.2%; *p* = 0.0053). These results suggest a strong association between JAK inhibitor therapy and the D2T-RA population. The observed relationship between JAK inhibitor use and controlled disease activity—measured by DAS28 and CDAI—further supports recent findings. In our cohort, CDAI values were similar in both groups (median 3, IQR 3–3.5), whereas DAS28 scores were slightly higher in D2T patients (median 1.67, IQR 1.5–3.2) compared to NO-D2T patients (median 1.56, IQR 1.5–3.1). Recent studies have demonstrated that up to 45% of D2T-RA patients achieve remission or low disease activity over five years with JAK or IL-6 inhibitors, underscoring the therapeutic potential of these agents in this challenging population [31]. Additional evidence supports a central role for JAK inhibitors in D2T-RA management, showing superior outcomes compared to TNFi, IL-6i, and CTLA-4Ig therapies during disease progression [32]. Further confirmation comes from Kanda et al. (FIRST Registry, 2025), who compared second-line b/tsDMARDs (TNFi, IL-6i, CTLA-4Ig, JAKi) and evaluated remission using the CDAI. JAK inhibitors demonstrated the greatest improvement in CDAI scores and the highest remission rates, with Upadacitinib achieving the most pronounced therapeutic efficacy [33].

## 5. Conclusions

In conclusion, our findings confirm several associated factors to D2T-RA, in line with current evidence. These include a lower proportion of male patients, longer disease duration, higher RF and ACPA titers, elevated ESR levels, greater corticosteroid exposure, more frequent use of advanced therapies (b/tsDMARDs), and a higher rate of treatment failure with these agents. Moreover, a relevant observation concerns the association between well-controlled clinical disease activity—measured by DAS28 and CDAI scores—and the use of JAK inhibitors, particularly Upadacitinib and Filgotinib, which were the predominant drugs in the D2T cohort. In contrast, other variables such as female sex, BMI, smoking, CRP, DAS28-ESR, CDAI, HAQ, PGA, PhGA, comorbidities, prednisone dose, MTX therapy, MTX dose, and monotherapy showed no significant differences between D2T and non-D2T patients, suggesting a limited predictive value in our population. Overall, these results emphasize the importance of early identification and tailored management of patients at risk for D2T-RA to prevent chronic refractoriness and optimize treatment outcomes. Future research should focus on elucidating the biological and molecular mechanisms underlying treatment resistance, thereby fostering the development of precision medicine strategies in RA.

## Figures and Tables

**Table 1:** Characteristics of the analyzed population.

Variable	NO D2T (*n* = 180; 52%)	D2T (n = 164; 48%)	*p*
Age (years)	70 (60.5–79)	69 (57–78)	NS
Female, n (%)	128 (71%)	143 (87%)	NS
Male, n (%)	52 (29%)	21 (13%)	**0.004**
Disease duration (months)	60 (20–114)	108 (84–120)	**0.0001**
BMI (kg/m^2^)	28 (26–28)	28 (26–29)	NS
Active smokers, n (%)	10 (5.5%)	6 (3.6%)	NS
Ex-smokers, n (%)	15 (8.3%)	11 (6.7%)	NS
RF (IU/mL)	46 (20–98)	64 (20–156)	**0.031**
ACPA (IU/mL)	108 (20–360)	178 (20–1600)	**0.0053**
ESR (mm/h)	9 (6–19)	18 (13–20)	**0.018**
CRP (mg/dL)	0.24 (0.09–0.56)	0.23 (0.05–0.7)	NS
DAS28-ESR	1.56 (1.5–3.1)	1.67 (1.5–3.2)	NS
CDAI	3 (3–3.5)	3 (3–3.5)	NS
HAQ	0.5 (0.5–0.75)	0.75 (0.5–0.75)	NS
PGA	3 (3–4)	3 (3–4)	NS
PhGA	3 (3–4)	3 (3–4)	NS
Hypertension, n (%)	63 (35%)	61 (37%)	NS
CV disease, n (%)	66 (36%)	51 (31%)	NS
Diabetes mellitus, n (%)	16 (8.8%)	12 (7.3%)	NS
Fibromyalgia, n (%)	13 (7.2%)	10 (6%)	NS
Depression, n (%)	15 (8.3%)	14 (8.5%)	NS
Interstitial lung disease, n (%)	10 (5.5%)	13 (7.9%)	NS
COPD, n (%)	9 (5%)	0	NS
Osteoporosis, n (%)	36 (20%)	40 (24.4%)	NS
CKD, n (%)	1 (0.5%)	2 (1.2%)	NS
Steroid treatment, n (%)	16 (8.8%)	28 (17%)	**0.028**
Prednisone dose (mg/day)	5 (2.5–5)	5 (3.7–5)	NS
Methotrexate treatment, n (%)	63 (35%)	33 (20%)	0.02
Methotrexate dose (mg/week)	10 (10–10)	10 (5–10)	NS
Monotherapy, n (%)	97 (53.8%)	107 (65%)	NS
Advanced therapy (b/tsDMARDs), n (%)	135 (75%)	163 (99.3%)	**0.0001**
Current DMARD, n (%)	45 (25%)	27 (16%)	NS

Baseline demographic, clinical, and therapeutic features of the total study population (n = 344), with comparison between patients with no-D2T RA and those with D2T RA. Statistically significant differences between groups are indicated (*p* < 0.05). Data are presented as median (IQR) or number (percentage). Abbreviations: RA, rheumatoid arthritis; D2T, difficult-to-treat; RF, rheumatoid factor; ACPA, anti-citrullinated peptide antibody; ESR, erythrocyte sedimentation rate; CRP, C-reactive protein; DAS28, Disease Activity Score for 28 joints; CDAI, Clinical Disease Activity Index; HAQ, Health Assessment Questionnaire; PGA, Patient Global Assessment; PhGA, Physician Global Assessment; BMI, body mass index; b/tsDMARDs, biological/targeted synthetic disease-modifying antirheumatic drugs; csDMARDs, conventional synthetic DMARDs; NS, not significant.

**Table 2:** Treatment differences with b-DMARDs and ts-DMARDs.

Advanced Therapy Failure	NO D2T	D2T	*p*
Etanercept	1.10%	28%	**0.00001**
Infliximab	0	5.50%	**0.0014**
Adalimumab	1.60%	20.10%	**0.00001**
Certolizumab	0	10.40%	**0.0001**
Golimumab	0	1.20%	NS
Tocilizumab	0	16.50%	**0.00001**
Sarilumab	0	4.80%	**0.0029**
Abatacept	1.10%	19.50%	**0.00001**
Rituximab	0	3.70%	**0.012**
Filgotinib	0	4.90%	**0.0029**
Upadacitinib	0	8.50%	**0.00043**
Tofacitinib	0	3.65%	**0.012**
Baricitinib	0	7.30%	**0.00017**

NS, not significant.

**Table 3:** The percentage of persistence across multiple treatment lines in the last 18 months before the evaluation.

Advanced Therapy Failure	NO D2T	D2T	*p*
Abatacept	14.00%	8.60%	NS
Adalimumab	4.00%	7.40%	NS
Baricitinib	2.20%	1.78%	NS
Certolizumab	4.40%	2.70%	NS
Etanercept	25.00%	14.70%	NS
Filgotinib	2.20%	10.50%	**0.015**
Sarilumab	13.00%	8.00%	NS
Tocilizumab	26.00%	28.00%	NS
Tofacitinib	3.00%	4.00%	NS
Upadacitinib	5.20%	13.00%	**0.0053**
Golimumab	0.00%	1.22%	NS
Rituximab	0.00%	0.60%	NS

NS, not significant.

**Table 4:** Logistic regression relationship between clinical and serological variables.

Variable	*p*	Odds Ratio (CI 95%)
Gender M/F	0.0017	0.377 (0.18–0.47)
Disease duration	0.0001	1.0065 (0.63–1.58)
ACPA	0.0009	1.0007 (0.64–1.69)
ESR	0.0157	1.02 (0.75–1.26)

**Table 5:** Multiple regression relationship between clinical and serological variables.

R^2^ = 0.68		
Variable	t	*p*
Gender M/F	3.27	0.012
Disease duration	4	0.0001
ACPA	2.9	0.036
ESR	2.38	0.017

## Data Availability

The raw data supporting the conclusions of this article will be available from the authors without undue reservation.
